# The Relationship between Face Processing, Cognitive and Affective Empathy

**DOI:** 10.3390/bs13010021

**Published:** 2022-12-26

**Authors:** Carmen Moret-Tatay, Paloma Mundi-Ricós, Tatiana Quarti Irigaray

**Affiliations:** 1 MEB Lab, Universidad Católica de Valencia San Vicente Mártir, Avenida de la Ilustración 2, Burjassot, 46100 Valencia, Spain; 2 ARIHA, Pós-Graduate Program in Psychology, Pontifícia Universidade Católica do Rio Grande do Sul, Porto Alegre 91215-330, Brazil

**Keywords:** face recognition, cognitive empathy, affective empathy

## Abstract

This study aims to examine the relationship between affective and cognitive empathy scores and perceptual face recognition skills. A total of 18 young adults participated in the study. Cognitive and Affective Empathy Test (TECA), The eyes Test and an experimental task were carried out. The experimental task has two blocks, a presentation, and a recognition phase, under the Karolinska battery of images expressing different emotions. Cognitive empathy sub-factors were found to be related to the hit rate on the recognition of surprise faces as well as the discarding of faces of disgust. In relation to the hit rate on discarding faces of disgust, this was related to perspective taking. Reaction time and Cognitive empathy subfactors were found to be positively correlated to the recognition of disgust, surprise, and sadness. Lastly, Perspective taking was also related to the discarding of disgust reaction time in a direct way. The relationships between affective empathy and other measures for emotional face recognition were not statistically significant. Knowledge of individual differences in cognitive and affective empathy, as well as of their relationship with behavioral responses such as the recognition or dismissal of emotional faces are of interest for social interaction and in psychotherapy.

## 1. Introduction

Empathy is a construct that has been addressed from multidimensional perspectives for decades [1,2]. One of the most popular distinctions has been pointed out between cognitive and affective empathy. The first refers to the ability to understand emotions, while the latter refers to the ability to share those emotions [3]. This distinction has found anatomical support, with different structures being differentiated for each dimension [4,5]. While the left anterior cingulate cortex has been related to cognitive empathy, the right anterior insula and the right inferior frontal gyrus seems to be more related to the affective, according to a meta-analysis [6].

As both definitions of empathy are considered latent constructs that cannot be directly assessed, this study is focused on behavioral response. While facial emotion recognition cannot be considered as a direct reflection of empathy, the literature has considered an overlap with emotional empathy in perceptual processing, as well as of cognitive empathy with inferential processes [7,8]. The literature also seems to indicate that highly empathetic individuals are more sensitive to human expressions, hypothesizing that highly-empathetic individuals might pay more attention than individuals with lower empathetic scores when discriminating facial expressions [9]. Some studies have questioned if trait empathy correlates with attention elicited by discriminating facial expressions, while others have indicated evidence of relationship between trait empathy and brain activation elicited by facial expressions [10].

According to the mirror neuron activation approach, for a proper empathic response, perceptual ability is necessary to process socially relevant stimuli, e.g., facial expressions [11]. Not surprisingly, individuals with higher affective empathy also seem to be particularly sensitive in reacting to facial expressions in an accurate way [12]. However, it has also been hypothesized that high affective empathy may interfere with the efficiency of visual recognition, causing personal distress [13]. In that case, cognitive empathy might be a more favorable resource. Cognitive empathy has sometimes been related to theory-of-mind or perspective-taking abilities [14]. However, when focusing on popular tools such as the eyes test [15] in the field, a relationship of this tool with affective measures has been described [16,17].

The indirect and probably most common way of measuring affective and cognitive empathy is through questionnaires. The revised *Reading the Mind in the Eyes* test is one of the most widely used tools. It is employed to assess emotion recognition, providing insight into the ability to recognize emotional expressions by determining an individual’s complex cognitive mental state from a partial facial expression. On this front, the literature has pointed out that facial emotion recognition partially mediated the relationship between reasoning by analogy and social cognition by employing the Reading the Mind in the Eyes Test [18]. With regards to the distinction between cognitive and affective empathy, an optimal strategy is to use the Cognitive and Affective Empathy Test [19], also known as TECA (*Test de Empatía Cognitiva y Afectiva*) [20] or the Interpersonal Reactivity Index [1]. Both tools are divided into four subscales, two of them assessing cognitive empathy (Perspective Taking and Emotional Understanding), the other two assessing affective empathy (Empathic Distress and Empathic Happiness).

By combining a questionnaire methodology with an experimental task, this study aims to examine the relationship between affective and cognitive empathy scores and perceptual face recognition skills under the cognitive and affective model proposed by Davis (1980;1983). Considering previous literature linking cognitive empathy to theory-of-mind [14], it is hypothesized that cognitive empathy scores are directly related to hit rates in a simple emotional face recognition task (hypothesis 1). Individuals with high cognitive empathy scores would show better perceptual and abstractive processing. Finally, it is hypothesized that affective empathy scores are directly related to reaction times (hypothesis 2). This would explain a higher cognitive cost of emotion processing in individuals with high affective empathy scores, as described in the previous literature [13].

## 2. Materials and Methods

### 2.1. Procedure and Participants

The study was conducted after obtaining ethical approval from the Universidad Católica San Vicente Mártir Ethics Committee (UCV2017-2018-31) and receiving participants’ written informed consent. Anonymous data was collected from young adults on the Southeastern coast of Spain in March 2022.

**Figure 1:**
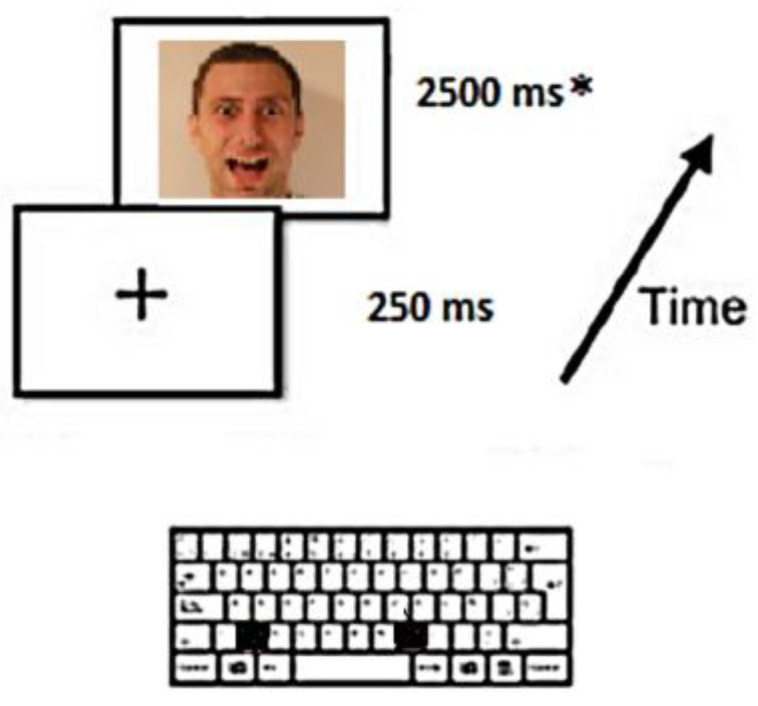
Task displays and trial structure for the matching task*. Each stimulus had a maximum duration for recognition of 2500 ms*. A simulation of KDEF images was employed because of copyright reasons.

Before conducting the experiment, participants were informed about the implications of the study and were asked to sign the written consent form. Participants were assessed in a silent room with a computer. For the first part, the face recognition task, on the screen they could observe two pictures of two people: a man and a woman with neutral expression, and six emotional facial expressions: anger, surprise, disgust, enjoyment, fear, and sadness [21]. Participants had to memorize the two faces in order to be able to recognize them afterwards. Furthermore, these figures could be expressing different emotions. As depicted in Figure 1, each time they recognized one on the picture, they had to tap the M keyboard key, and each time they were presented with a picture of someone else, they had to press the Z. Due to the copyright of the KDEF images, they have been simulated in Figure 1. The DMDX software [22] was employed for image presentation.

A total of 18 young adults participated in the study. Sex was controlled across the sample (50% were women and 50% men) with ages ranging from 25 to 30 years. In terms of inclusion criteria, all the participants had normal or corrected to normal vision, were native Spanish speakers and did not report cognitive or neurological disorders. The participants were chosen after a personal interview.

### 2.2. Measures

After a sociodemographic battery, individual differences in empathic tendencies were evaluated using the Cognitive and Affective Empathy Test (TECA), which consists of 33 questions, with Likert-type responses ranging from 1 (strongly disagree) to 5 (strongly agree). It allows the measurement of both cognitive empathy and emotional empathy. Regarding the validity of the TECA, it has shown strong convergent validity, with high correlations with the Spanish adaptation of the Interpersonal Reactivity Index [23].

The eyes test (Baron-Cohen et al., 2001) was used to assess cognitive empathy. It consists of 36 slides with pictures of facial expressions on the eye line only. Each sheet contains four adjectives from which the participant must choose that that best describes the expression in the image. The adjectives correspond to complex emotional states such as bored or arrogant, rather than simple emotional states such as sad or happy. The test is corrected by assigning one point for each correct answer, so the score varies from 0 to 36.

Lastly, the Karolinska battery of images expressing different emotions was employed for the experimental task [24]. The task has two blocks, a presentation, and a recognition phase. Thus, there was a total of 392 items per participant (or 28 items per 7 conditions, where 14 were a man a 14 a woman); 192 in the presentation and 392 in the recognition block (192 targets and 192 distractors divided into 7 face emotions). The emotions selected as stimuli, as described in previous literature, were fear, sadness, happiness, anger, surprise, and disgust, besides another neutral image. The DMDX software was employed [22]. This is a display system that carries out the randomization of the images’ presentation and records reaction time of the participants to respond to the stimuli presented.

### 2.3. Design and Statistical Analysis

This study uses a combination of experimental and survey method. G*Power [25] was employed to calculate the sample size. A medium effect for an experimental paradigm suggested a minimum of 18 participants. Stimuli in the experiment were counterbalanced to diminish progressive error. This allowed us to examine any possible biases related to the stimuli characteristics. For example, one of the images has characteristics which people remember better than the others. It should be noted that we did not find statistically significant differences in terms of list of presentation (*p* > 0.05). A cut-off technique was applied for reaction time lower than 250 ms and higher than 1500, as described in previous literature [26,27,28,29], trimming 3% from the whole data set. Data analysis was performed using JASP (Version 0.12.2).

## 3. Results

Results were examined in terms of accuracy (hypothesis 1) and response latency (hypothesis 2). Table 1 describes the whole scores on the variables under study. Differences across participant’s sex were examined using Mann-Whitney U test. No statistically significant difference was found (*p* > 0.05), though Reaction Time (RT) across distraction and target images related to each other in a positive way, and Empathic Distress (ED) and Empathic Happiness (EH) appeared to be in the same direction. When Spearman’s rho coefficients were carried out to check for accuracy of each emotion (hits) and the other questionnaire variables, a positive correlation between Emotional Understanding (EU) and surprise for target images was found (rho = 0.528; *p* < 0.05). Moreover, a positive correlation between Perspective Taking (PT) and disgust for distracting images was found (rho = 0.494; *p* < 0.05).

Secondly, when the same analysis was carried out for RT across each emotion and the other questionnaire variables, a positive correlation between PT and disgust for target images was found (rho = 0.473; *p* < 0.05), as well as sadness for target images (rho = 0.570; *p* < 0.05). PT showed a positive relationship with surprise for target images, but this result did not reach statistically significant level (rho = 0.469; *p* = 0.05). With regards to the distracting stimuli, there was a positive correlation between PT and disgust (rho = 0.526; *p* < 0.05).

Lastly, linear multiple regression models, were carried out. Thus, the scores for eyes test and TECA were entered as the predictors and the outcome variables were the seven stimuli conditions for emotional faces (neutral, fear, sadness, happiness, anger, surprise and disgust) in the target and distracting condition across accuracy and RT dependent variables. None of these models reached statistically significant levels except for the model on the prediction of surprise emotion for the accuracy on target images. In this case the EU depicted the following values: β = 0.66; *p* < 0.05.

## 4. Conclusions and Discussion

The aim of this study was to examine the relationship between affective and cognitive empathy scores with emotional face recognition in university students. For this purpose, an experimental task of emotional face recognition, combined with questionnaires measuring cognitive and affective empathy (TECA), and the eyes test, which measures individual differences in theory of mind capabilities and more precisely emotion perception [16,17], were used in a university sample of 18 participants. Cognitive empathy sub-factors were found to be more related to the hit rate on recognition of surprise faces and the discarding of disgust faces. The hit rate measure for distracting disgust faces was positively related to perspective taking. Reaction time to Cognitive empathy subfactors were found to be related to recognition of disgust, surprise and sadness. Finally, Perspective taking was again related to the discarding of disgust reaction time. The relationships between affective empathy and other measures for emotional face recognition were not statistically significant.

According to the first hypothesis, current results support that cognitive empathy scores are directly related to hit rates for recognition of surprise and disgust facial expressions. Individuals with high cognitive empathy scores show higher accuracy for disgust and surprise faces. However, this was not the case for affective empathy. The findings of this study aligns with the definition of empathy as a cognitive dimension in which one person attempts to comprehend the feelings, thoughts, or intentions of another person [30]. However, when individuals with high emotional reactions are tested with TECA and eyes test, affective measure does not seem to benefit from this strategy.

The results of the second hypothesis does not show any interference in reaction times or cognitive cost for individuals with higher affective empathy scores. Thus, current results seem to be inconclusive for the second hypothesis. One of the limitations that might explain this result could be related to the use of instruments under study. Although TECA and IRI measures the same constructs, there are differences between the sub-factors of both scales. Previous literature using IRI found affective empathy to have opposite relationships with the recognition of facial expressions of emotions. Specifically, the authors have described that empathic concern was positively related, while personal distress was negatively related, to accurate emotion recognition. However, the present results do not support this finding with the TECA and eyes test.

Moreover, the sample size is one of the limitations of the of the present study. While experimental studies with repeated measures can be used with smaller numbers of participants, future studies should consider larger a samplesl using survey techniques.

The novelty of this study lies in the analysis of emotional face recognition and the study of the empathy relationship under the Davis model (1980, 1983). These results might be of interest for interventions to help recognize emotions and improve social interactions. Knowing that there are individual differences in cognitive and affective empathy, as well as in their relationship with behavioral responses such as the recognition or dismissal of emotional faces, the results could be connected to aspects of social interaction and psychotherapy.

## Figures and Tables

**Table 1:** Descriptive Statistics of the variables under study. The numbers 1–8 are the variables in the first column.

	Mean	*SD*	Min.	Max.	1	2	3	4	5	6	7	8
1. Accuracy	0.979	0.015	0.941	0.997	—							
2. Target (RT)	616.331	89.183	483.430	816.026	0.158	—						
3.Distracting (RT)	615.361	78.671	471.664	773.984	0.158	0.950 **	—					
4. Eyes test	24.056	3.702	15.000	31.000	−0.191	−0.073	−0.115	—				
5. PT	21.556	2.706	18.000	28.000	0.381	0.327	0.341	0.125	—			
6. EU	20.611	2.404	16.000	25.000	0.370	−0.061	−0.018	0.025	0.376	—		
7. ED	26.167	1.790	23.000	30.000	0.296	−0.186	−0.107	−0.041	0.202	0.431	—	
8. EH	19.444	1.854	15.000	22.000	0.287	−0.232	−0.195	0.097	0.249	0.434	0.617 **	—

Note. Target and Distracting are related to the global Reaction Times (RT). PT = Perspective Taking; EU = Emotional Understanding; ED = Empathic Distress; EH = Empathic Happiness. ** = *p* < 0.01.

## Data Availability

The data presented in this study are available on request from the corresponding and second author.
